# Anesthesia Approach to Managing Severe Hemorrhagic Shock and Anemia With Non-transfusion Alternatives in a Practicing Jehovah's Witness: A Case Report

**DOI:** 10.7759/cureus.53301

**Published:** 2024-01-31

**Authors:** Colin Kirsch, Romain Rabany, Matthew Pon, Julia Shabanian, Anand Narayanappa

**Affiliations:** 1 Medical School, Creighton University School of Medicine, Phoenix, USA; 2 Anesthesiology, Creighton University School of Medicine, Phoenix, USA

**Keywords:** shared decision-making, patient-centered care, non-transfusion therapy, medical ethics, patient autonomy, severe anemia, jehovah's witness, blood transfusion, trauma, hemorrhagic shock

## Abstract

Traumatic hemorrhagic shock is a common yet life-threatening occurrence across the United States and is typically managed with blood transfusions as the standard of care. However, providers caring for a Jehovah's Witness patient who refuses transfusions due to religious reasons face unique ethical challenges in upholding evidence-based shock resuscitation protocols while respecting the patient's autonomy and faith-based stance that strictly prohibits blood products. We present a complex clinical case of a 46-year-old Jehovah's Witness who developed severe hemorrhagic shock, partial amputation, and critical anemia after a traumatic 40-mile-per-hour motorcycle collision resulting in comminuted fractures and arterial disruption. Despite receiving emergent blood transfusions initially, further transfusions were declined once his identity as a practicing Jehovah's Witness was disclosed. His hemoglobin plunged to dangerously low levels of 4.6 g/dL before stabilizing to 5.3 g/dL with pharmaceutical alternatives including intravenous iron, high-dose erythropoietin, and phlebotomy minimization. Respecting patient convictions while delivering effective evidence-based shock management created significant ethical conflicts given the proven efficacy of blood transfusions. However, this complex case demonstrates that through meticulous medical and surgical care coordinated by a multi-disciplinary team applying customized non-transfusion techniques, traumatic hemorrhagic shock and life-threatening anemia can still achieve favorable outcomes without relying on transfusions when respecting faith-based refusal of blood products.

## Introduction

Traumatic hemorrhagic shock is a severe and life-threatening condition requiring urgent resuscitation, often with blood product transfusions to maintain adequate circulation and oxygen delivery to vital organs. Up to 5% of civilian trauma patients require massive transfusions with the goal of improving survival mainly by increasing the platelet-to-red blood cell (RBC) ratios [1]. Massive blood loss leads to dangerously low blood pressure and cellular shock, which can rapidly progress to multi-organ failure and death without prompt correction. First-line management focuses on rapidly controlling external bleeding sources along with initiating damage control resuscitation protocols using packed RBCs (PRBCs), fresh frozen plasma (FFP), platelets, and coagulation factors or fibrinogen concentrate to restore circulating blood volume, clotting factors, and oxygen-carrying capacity [2].

However, some patients object to receiving blood products due to religious beliefs, such as Jehovah's Witnesses who refuse transfusions of whole blood or its primary components. This presents an ethically challenging situation for medical teams to provide standard, potentially life-saving care while respecting patients' autonomy and wishes [3]. Management requires a balanced multi-disciplinary approach adhering to restrictive transfusion protocols and evidence-based guidelines utilizing pharmaceutical alternatives to stabilize and ameliorate severe anemia and physiologic derangements from shock. We present a complex case of a Jehovah's Witness with hemorrhagic shock and severe anemia following a traumatic injury.

## Case presentation

A 46-year-old male Jehovah's Witness presented to the emergency department following a high-speed 40-mile-per-hour motorcycle collision resulting in severely comminuted right lower extremity fractures with impending amputation with arterial disruption and uncontrolled hemorrhagic shock. The patient lost a significant amount of blood volume at the trauma scene, where a field tourniquet was applied in an attempt to slow ongoing severe external bleeding.

On arrival at the hospital, his systolic blood pressure plunged to the 90s mmHg, and signs of cellular shock were present, including tachycardia, cool extremities, altered mental status, and delayed capillary refill. A massive transfusion protocol was urgently initiated to provide circulating volume, perfuse end organs, and attempt to achieve hemodynamic stability. After receiving four units of PRBCs, two units of FFP, and one unit of platelets, his hemorrhagic shock temporarily resolved. He subsequently underwent an emergent right lower extremity wound debridement and washout procedure, along with the placement of an external fixator to stabilize bony fractures. Anesthesia utilized standard induction agents, including 20 mcg etomidate, 100 mg rocuronium, and 50 mg meperidine, along with sevoflurane gas. Intraoperative propofol infusions and a total of 200 mcg of fentanyl were utilized for the maintenance of general anesthesia. Surgical hemostasis was achieved, and the patient was admitted to the surgical intensive care unit for close monitoring and resuscitation.

On postoperative day 2, the patient disclosed to the team that he was a practicing Jehovah's Witness, precluding any further blood product transfusions given his religious convictions against receiving whole blood or its primary components. His hemoglobin had decreased to 6.8 g/dL following the trauma and subsequent blood loss, but he remained hemodynamically stable without the need for vasopressor medications to maintain his blood pressure. However, by postoperative day 3, his hemoglobin had dropped even further to a critically low level of 4.6 g/dL, triggering consultations with hematology specialists. The hematology team recommended medically managing his declining hemoglobin and severe anemia by starting intravenous iron supplementation to increase iron stores and high-dose erythropoietin injections to stimulate RBC production and minimizing any unnecessary phlebotomy to limit further iatrogenic blood loss from lab draws. With these interventions, the patient was stabilized to a hemoglobin level of 5.3 g/dL, which remained low but was deemed sufficient to postpone any further non-emergent surgeries.

On hospital day 11, the patient underwent a complete right above-knee amputation procedure at a stable hemoglobin of 5.3 g/dL. The anesthesiologist used 2 mg midazolam, 75 mg lidocaine, and 150 mg propofol for induction, with propofol infusions and sevoflurane gas used for maintenance. Intraoperatively, blood loss was kept to a minimum with the utilization of a pneumatic tourniquet and advanced electrocautery devices. As recommended by the consulting hematology team, all forms of anticoagulation and chemical thromboprophylaxis were held due to concerns over bleeding risks exacerbating the patient's severe anemia.

In the postoperative recovery phase, the patient later developed a symptomatic left lower extremity peroneal deep vein thrombosis (DVT). The patient was started on apixaban, a direct oral anticoagulant, and the DVT was monitored with duplex ultrasound and remained stable. The patient's hemoglobin gradually improved to surpass 7 g/dL over the ensuing days, signaling bone marrow recovery. His erythropoietin stimulation injections were similarly adjusted from high-dose 40,000 units daily to 40,000 units weekly moving forward. On hospital day 17, he was determined to be medically stable for discharge to an acute rehabilitation facility with a plan for continued outpatient erythropoietin injections, oral apixaban therapy, iron supplementation, and close monitoring.

## Discussion

**Figure 1:**
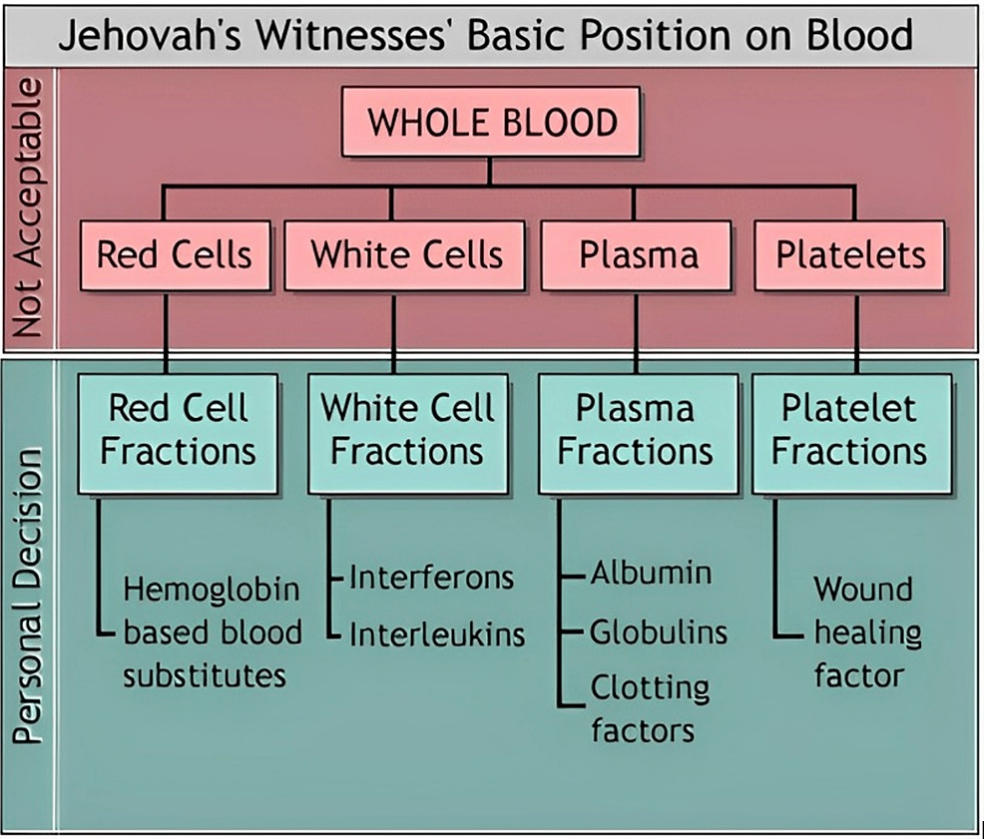
Jehovah's Witnesses' basic position on blood Reference: [4]

The ethical management of a Jehovah's Witness patient declining blood transfusions after major trauma, hemorrhagic shock, and severe anemia poses several clinical challenges. Physicians must aim to uphold evidence-based standards of care in stabilizing patients while seeking to respect patient autonomy and understanding individual preferences regarding their medical treatment. Nearly all Jehovah's Witnesses do not accept red cells, platelets, white cells, and unfractionated plasma. Many Jehovah's Witnesses accept derivatives of primary blood components such as albumin solutions, cryoprecipitate, clotting factor concentrates, and immunoglobulins. It is a personal decision to accept and receive these derivatives, and it must not be assumed that all Jehovah's Witnesses will accept them (Figure 1).

In the setting of traumatic hemorrhagic shock, massive transfusion protocols administered in a restrictive strategy have demonstrated reduced 30-day mortality [5]. However, refusal of blood product transfusions despite life-threatening clinical scenarios often creates conflict, given their proven efficacy and historical role as a cornerstone of trauma and critical care management [6]. The Health Care Consent Act states that "a treatment may be given without consent in an emergency" if, in the physician's opinion, there is no means of communication with the patient and if the delay might place the patient in sustaining severe bodily harm [7]. This can be seen in our case, with the patient receiving blood products in an emergent scenario before it was known they were a Jehovah's Witness. However, many Jehovah's Witnesses have their blood cards on them or their wishes documented, leaving physicians in the same initial dilemma. Courts have upheld charges of assault against physicians who ordered the administration of a blood transfusion to a patient, explicitly declining blood transfusion on religious grounds [8]. Thus, knowing alternative methods to manage hemorrhagic shock and severe anemia in this population is paramount.

Effective non-transfusion methods to medically stabilize and correct severe anemia include but are not limited to iron supplementation, exogenous erythropoietin, polymerized hemoglobin (PolyHeme, Northfield Laboratories, Evanston, Illinois, United States), methylene blue, minimizing unnecessary phlebotomy, and deferring non-emergent surgeries to be done once anemia improves [9,10]. Intravenous iron supplementation is needed to increase iron stores needed for erythrocyte production, along with erythropoietin injections to stimulate RBC generation. Minimizing unnecessary phlebotomy preserves circulating blood volume while topical hemostatics, perioperative, tourniquets, electrocautery devices, and careful surgical techniques reduce intraoperative blood losses. Individualized thromboprophylaxis regimens can balance thrombosis and bleeding risks. Once a patient is stabilized, managing subsequent care requires vigilant treatment to avoid patient decline while the patient's bone marrow compensates for their anemia. Our case exemplifies how the judicious application of multiple blood-conserving modalities permits successful outcomes without reliance on transfusions.

Anesthetic selection requires thorough attention to detail to ensure cardiovascular stability, given limited reserves, reducing blood loss. Agents utilized here, such as etomidate, sevoflurane, and propofol, provide reliable induction, maintenance, and rapid emergence from anesthesia for the hemorrhagic shock patient while respecting religious objections.

## Conclusions

Managing hemorrhagic shock in Jehovah's Witness patients declining transfusions requires balancing evidence-based resuscitation with respecting patient autonomy. Effective non-transfusion techniques include iron supplementation, exogenous erythropoietin, polymerized hemoglobin (PolyHeme), methylene blue, minimizing unnecessary phlebotomy, customized thromboprophylaxis, and postponing non-emergent surgeries. Our case shows that with coordinated care applying pharmacological alternatives for severe anemia and shock, trauma teams can optimize care for Jehovah's Witnesses without transfusions. Different patient comorbidities and American Society of Anesthesiologists (ASA) statuses further complicate medical treatment. Our case identifies a possible baseline treatment plan for subsequent non-emergent surgeries at a hemoglobin of 5.3 g/dL for patients who decline blood transfusions. In this setting, we demonstrate the safe use of midazolam, sevoflurane, and propofol for anesthesia with concurrent intraoperative measures to reduce bleeding. Further research on patient outcomes and quality of life using these modalities in Jehovah's Witness patients is warranted. Open communication and shared decision-making are key in aligning values, judgments, and ethical duties.
